# UK adult ADHD services in crisis

**DOI:** 10.1192/bjb.2023.88

**Published:** 2024-02

**Authors:** Michael C. F. Smith, Raja A. S. Mukherjee, Ulrich Müller-Sedgwick, Dietmar Hank, Peter Carpenter, Marios Adamou

**Affiliations:** 1 Leeds and York Partnership NHS Foundation Trust, Leeds, UK; 2 Surrey and Borders Partnership NHS Foundation Trust, Redhill, UK; 3 Le Bas Centre, St Helier, Jersey; 4 University of Cambridge, Cambridge, UK; 5 Avon & Wiltshire Mental Health Partnership NHS Trust, Bath, UK; 6 University of Bristol, Bristol, UK; 7 Royal College of Psychiatrists, London, UK; 8 South West Yorkshire Partnership NHS Foundation Trust, Wakefield, UK; 9 University of Huddersfield, Huddersfield, UK

**Keywords:** ADHD, diagnosis, quality of care, service delivery, attention-deficit hyperactivity disorder

## Abstract

The UK's services for adult attention-deficit hyperactivity disorder (ADHD) are in crisis, with demand outstripping capacity and waiting times reaching unprecedented lengths. Recognition of and treatments for ADHD have expanded over the past two decades, increasing clinical demand. This issue has been exacerbated by the COVID-19 pandemic. Despite an increase in specialist services, resource allocation has not kept pace, leading to extended waiting times. Underfunding has encouraged growth in independent providers, leading to fragmentation of service provision. Treatment delays carry a human and financial cost, imposing a burden on health, social care and the criminal justice system. A rethink of service procurement and delivery is needed, with multiple solutions on the table, including increasing funding, improving system efficiency, altering the service provision model and clinical prioritisation. However, the success of these solutions hinges on fiscal capacity and workforce issues.

Adult attention-deficit hyperactivity disorder (ADHD) waiting lists pose a significant national problem,^1,2^ an issue unlikely to resolve without substantial intervention. Over the past two decades, recognition^3^ and effective medical treatments^4^ for ADHD have markedly expanded, contributing to a consistent increase in clinical demand across the UK.^5^ Notably, the consensus among authors is that the COVID-19 pandemic has further exacerbated the issue of waiting times.

During the mid-1990s, only a handful specialist adult ADHD services existed in the UK, often composed of small, resource-limited teams. These teams were tasked with addressing extensive regional clinical demands for assessments and treatments. Following the publication of the 2008 National Institute for Health and Care Excellence (NICE) guidelines, specialist services throughout England significantly increased.^6^ Despite this growth, many regions of the UK still experience limited access to adult ADHD services.^7^

In addition to this, the resources allocated to these services have failed to keep pace with the ever-growing demand, resulting in lengthy waiting lists for specialist services. A 2018 Freedom of Information request to clinical commissioning groups highlighted a substantial variation in waiting times for assessment following referral, ranging from 4 weeks to nearly 4 years.^1^

Statistics concerning waiting lists can be misleading. Headline figures usually consider the individual at the front of the queue. However, for an individual awaiting assessment, the estimated waiting time from their addition to the list, or the ‘back of the queue’, is more relevant. For a service that currently quotes a 2–4 year wait from referral to assessment, a more realistic estimate for a newly added individual is likely to be 5–10 years if no additional funding is provided. This figure is based on calculations done by specialist services where demand is assumed to be static and clinical teams are operating at full capacity.

Although specialist services, the medical literature,^2^ the media^8^ and government^9^ increasingly recognise the widening gap between resource demand and capacity, a lack of both national strategy and political focus exacerbates this problem. The escalating problem of ADHD waiting times has continued largely unchecked owing to a lack of specific targets for these waiting times^10^ and the absence of routinely collected national data. This stems primarily from a lack of set targets for ADHD, as exist for other conditions, such as autism spectrum disorder.^11^ This issue was highlighted in the government's response to a parliamentary debate held in February 2023 on ADHD waiting times.^12^ The response concluded that, in the absence of a specified NICE target, local commissioning organisations, rather than central government, should resolve the problem of waiting lists.

According to the National Health Service (NHS) constitution,^13^ patients have a right to start consultant-led treatment within a maximum of 18 weeks of referral. However, the government does not consider this time frame applicable to ADHD.^12^ In response to the unmet standard following the COVID-19 pandemic, NHS England outlined its strategy for increasing capacity in its Elective Recovery Plan.^14^ This ambitious plan aimed to eliminate waits longer than 2 years by July 2022 and waits longer than 1 year by March 2025. However, this plan mainly addresses non-urgent hospital treatment, primarily surgical, and makes no reference to mental health services.

Underfunding of NHS adult ADHD services has resulted in a significant growth of independent providers, whose numbers have increased in response to the unmet demand. The National Health Service Commissioning Board and Clinical Commissioning Groups (Responsibilities and Standing Rules) Regulations 2012,^15^ embedded in the NHS Constitution, grant individuals living in England the right to choose the location of their first out-patient appointment with any provider that holds an NHS-commissioned contract. In view of this, many patients have accessed ADHD services through public funding via alternative routes.

Although this increased capacity has been welcomed by many patients facing long waits, it has also introduced additional challenges into the system. The large number of providers in both the NHS and the independent sector presents a challenge to patients, clinicians and commissioners tasked with navigating a complex and fragmented system and determining the quality and reliability of these services. Although NICE provides guidance on the assessment and treatment of ADHD^10^ it is not specific enough on what constitutes quality assessment and treatment to ensure the required consistency between providers. Financial and human resources have been diverted from NHS services,^16^ and duplication and wastage have resulted from NHS services needing to reassess people diagnosed elsewhere when there is a lack of confidence in assessments. Furthermore, many patients have been left without medication when their general practitioner (GP) has felt unable to continue ADHD medication under shared care arrangements because of a lack of confidence in the diagnosis. The General Medical Council^17^ places a clear responsibility on prescribers to prescribe medicine only where ‘you have adequate knowledge of the patient's health and are satisfied that the medicine or treatment serve the patient's needs’. These issues have recently been the focus of significant media attention following a BBC Panorama documentary.^18^

The extraordinarily long waiting times for ADHD services are particularly alarming given the effectiveness of treatments for this condition. Stimulant medications used for ADHD, which have one of the largest effect sizes in psychiatry,^19^ improve both symptom control and functional impairment,^20^ and are generally safe and well tolerated.^21^ Moreover, the need for timely treatment is underscored by the range of negative outcomes for individuals with the condition, including accidents/unintentional injuries,^22^ comorbidity,^23^ increased mortality^24^ and suicide.^25^ In addition, there is a financial cost to the individual,^26^ their family and the wider public ^27^ resulting from delayed access to treatment.

The human and financial costs of untreated ADHD represent a compelling case for investment in this area, considering that effective long-term management benefits both the individual and the wider economy. The impact of unaddressed ADHD reverberates across the broader system, affecting areas such as social care,^28^ health and the criminal justice system.^29^

ADHD in adults is a chronic condition with a substantial cost to society, requiring sustained and targeted investment to support those affected. Existing approaches to funding and delivery of services in the UK have been largely insufficient, as evidenced by the current state of the NHS services.

In conclusion, the crisis in adult ADHD services is an opportunity to rethink how these services are procured and delivered. The shortcomings of the current system highlight the need for investment, but more than that, they underscore the need for a different approach to service delivery.

## The road ahead

A range of solutions exists to address the crisis in adult ADHD services. However, the challenge lies in the fiscal and workforce problems. A multi-pronged approach is likely to yield the most substantial improvements.

### Option 1: status quo

Maintaining the current system is the first option. However, without changes, waiting lists will likely grow year after year, exacerbating the existing problems. This option is not only directly detrimental to patients but also imposes a significant burden on specialist services.

Lengthy waits for diagnosis create significant inefficiency in the system. Specialist services are expending considerable time in triaging requests for patients to be prioritised owing to worsening personal situations and liaising with patients who are complaining about the wait. The proportion of time devoted to this increases in tandem with the waiting list, which challenges the ability to engage in the core work of assessment and treatment. GPs are also expending valuable time supporting those waiting for care and corresponding with specialist services. The unmet need is also placing pressure on other services, such as secondary mental healthcare, owing to the burden of comorbid difficulties, such as anxiety, depression and the need for crisis support, that occur when the condition remains untreated. Partner agencies such as housing, social support and the criminal justice system will also continue to feel the effect of unmet need as the condition manifests in these areas.

The pressure of extended waiting times is also damaging morale within specialist services, bringing additional challenges of workforce retention and capacity, further exacerbating the problem.

Quality of care is also being threatened, with many services struggling to provide safe treatment and failing to meet the NICE standards of care where annual medical reviews are concerned.

Consequently, this approach is unlikely to be sustainable in the long run.

### Option 2: enhanced funding

The most direct solution is to maintain the current ADHD services model while substantially increasing funding. However, given the fiscal environment, this option seems unlikely. Furthermore, substantial funding would be required to bridge the gap between demand and capacity and to tackle the backlog.

### Option 3: improve system efficiency

Improving system efficiency could help tackle the crisis. This could be achieved by refining every aspect of the diagnostic and treatment pathway, including leveraging technology.

Quantified behavioural tests, which aim to provide a computerised objective assessment of ADHD symptoms, offer promise but require further investigation before they can be considered as a strategic solution.^30,31^ Artificial intelligence/machine learning approaches^32^ might help considerably in reducing the time taken to establish a diagnosis but again such developments are unlikely to affect the system quickly enough. As things stand, ADHD continues to require a clinical diagnosis, which can be aided but not made by a stand-alone technology.

Efficiency savings are possible using more sophisticated administrative systems for assessment and treatment. Such systems can streamline the process and have the potential to significantly reduce the administrative burden on specialist teams. The use of electronic prescribing also offers promise but is not available to secondary care and specialist teams in most areas.

Significant inefficiencies are introduced when patients move between different providers. In cases where the quality of the diagnostic report is insufficient to enable a provider to confirm and continue care a patient may be required to undergo a repeat assessment. This problem could be addressed by agreed standards for diagnostic reports.

Although promising, efficiency savings are unlikely to significantly alleviate the burden without accompanying efforts.

### Option 4: alter the service provision model

Another potential strategy is changing the ADHD service provision model. Instead of relying on specialist services for assessment and management, primary or secondary care could take up a larger portion of the workload.^6^ More ‘complex’ and risky patients could be assessed and treated by ‘case-holding’ teams, if they are already on the case-load (e.g. community mental health, perinatal and forensic teams). This approach would make better use of specialist resources but would require significant funding, training and implementation time. Given that ADHD is such a common condition, sole responsibility cannot rest with specialist services alone.

### Option 5: clinical prioritisation

A more controversial option is to limit access to services to those most severely affected by ADHD. This approach would raise ethical concerns and would pose significant practical challenges. A recent pilot to explore the viability of such an approach^33^ has received criticism.^34^

Rationing within the NHS is typically used when the threshold in question is relatively simple and easily measurable, for example weight for bariatric surgery or visual acuity for cataract surgery. Rationing within ADHD services via symptom severity, impairment level, risk or a combination of all three would be challenging owing to the subjective nature of the responses to the scales and a concern that some patients might exaggerate reporting to meet the threshold. Furthermore, specific patient groups, potentially those with the greatest needs, may struggle to comply with such processes and may be further disadvantaged.

### Summary of recommendations

- Agreed standards on diagnostic assessments and reports could improve consistency and allow for more efficient management across providers.
- A national target for ADHD waiting times might help better define and focus the impact of waiting times and stimulate positive change.
- A national strategy for ADHD would assist in raising the profile of the problem and supporting commissioning decisions.
- Additional funding is urgently required to enable adult ADHD services to better meet demand.

## Conclusions

Adult ADHD services in the UK are at a critical juncture. With increasing demand and insufficient resources, the current system is struggling to provide adequate care for patients. Alternatives that combine efficient administrative systems, alternative service models and routine data collection for political focus might offer some relief. However, significant strides will be made only with additional funding and a shift in how we approach ADHD treatment. The introduction of a national target for starting diagnostic assessments within 3 months of referral could stimulate positive change. Such a target could be integrated into the NICE guidelines, helping to keep ADHD treatment at the forefront of health policy.

## Data Availability

Data availability is not applicable to this article as no new data were created or analysed in its preparation.

## About the authors

**Michael Smith**, MSc, is a consultant psychiatrist and clinical lead of the adult ADHD service, Leeds and York Partnership NHS Foundation Trust, Leeds, UK. **Raja A. S. Mukherjee**, PhD, is a consultant psychiatrist in adult learning disability in the Adult Neurodevelopmental Team, Surrey and Borders Partnership NHS Foundation Trust, Redhill, UK. **Ulrich Müller-Sedgwick**, PhD, is a consultant psychiatrist and the Government of Jersey's lead for adult neurodevelopmental pathways, working with Adult Mental Health, Health and Community Services at Le Bas Centre, St Helier, Jersey, and a visiting research fellow in the Department of Psychiatry at the University of Cambridge, UK. **Dietmar Hank**, MSc, is a consultant psychiatrist and clinical lead of the adult ADHD service, Avon & Wiltshire Mental Health Partnership NHS Trust, Bath, UK. **Peter Carpenter**, MBChB, is an honorary lecturer in the School of Social and Community Medicine, University of Bristol, and Chair of the Neurodevelopmental Psychiatry Special Interest Group at the Royal College of Psychiatrists, London, UK. **Marios Adamou**, PhD, is a consultant psychiatrist and clinical lead of the adult ADHD service, South West Yorkshire Partnership NHS Foundation Trust, based at the School of Human and Health Studies, University of Huddersfield, UK.
